# Comparative analysis of *in situ* versus *ex situ* perfusion on flow and microcirculation in kidney procurement: research on a porcine model

**DOI:** 10.1186/2047-1440-2-13

**Published:** 2013-07-09

**Authors:** Daniel Foltys, Moritz Kaths, Mari Strempel, Uwe Scheuermann, Axel Heimann, Veronika Weyer, Torsten Hansen, Oliver Kempski, Gerd Otto

**Affiliations:** 1 Department of Transplantation and Hepatobiliopancreatic Surgery, University Medical Centre, Johannes Gutenberg University, Langenbeckstraße 1, 55131, Mainz, Germany; 2 Institute of Neurosurgical Pathophysiology, University Medical Centre, Johannes Gutenberg University, Langenbeckstraße 1, 55131, Mainz, Germany; 3 Institute of Medical Biostatistics, Epidemiology and Informatics, University Medical Centre, Johannes Gutenberg University, Langenbeckstraße 1, 55131, Mainz, Germany; 4 Institute of Pathology, University Medical Centre, Johannes Gutenberg University, Langenbeckstraße 1, 55131, Mainz, Germany

## Abstract

**Background:**

The first crucial step in transplantation appears to be the effective rinsing of the graft during organ procurement. Even though there is strong suspicion that *ex situ* perfusion results in better rinsing of the graft, there is no proof for this hypothesis. The aim of this study was to analyse the differences of *in situ* and *ex situ* kidney perfusion in a porcine model.

**Methods:**

Standardised multiorgan procurement was performed in 15 German landrace pigs. Perfusion was carried out using histidine–tryptophan–ketoglutarate solution (HTK) under the application of pressure. In one kidney, *in situ* perfusion via the aorta was carried out while the second kidney received *ex situ* perfusion via the renal artery (RA). Perfusate flow inside the aorta and the RA was recorded at different pressure steps. In order to visualise the effect on the microcirculation, different coloured microparticles (MPs; 10 μm) were administered via the aorta or RA. Subsequently, frozen sections of the explanted kidneys were analysed histologically and MPs were evaluated quantitatively.

**Results:**

*Ex situ* kidney perfusion resulted in significantly improved flow rates (*P*<0.0001) compared with *in situ* perfusion. By applying *ex situ* perfusion it was even possible to attain physiological flow levels on the RA under the application of external pressure of 150 to 200 mmHg. The amount of MPs was able to highlight the positive impact of *ex situ* perfusion on microcirculation of the kidney graft (*P*<0.0001).

**Conclusions:**

The use of MPs represents a valuable tool for quantitative investigation and illustration of kidney perfusion in experimental setups. Additional e*x situ* perfusion is able to improve the quality of kidney perfusion.

## Abstract (de)

**Abstract in German language:**

**Hintergrund**

Der erste und essentielle Schritt für eine Organtransplantation ist die effektive Spülung des Organs im Rahmen der Organentnahme. Obwohl es einige Hinweise dafür gibt, dass eine ex *situ* Perfusion die Spülung des Organs verbessern würde, gibt es bisher keinen Beweis für diese Hypothese. Das Ziel dieses Versuches war es daher die Unterschiede zwischen *ex situ* und *in situ* Perfusion eines Nierentransplantates in einem Großtiermodell zu untersuchen.

**Methodik**

Eine standardisierte Multi-Organentnahme wurde an 15 Schweinen Deutscher Landrasse durchgeführt. Als Perfusionslösung wurde Histidin-Tryptophan-Ketoglutarat (HTK) verwendet. Auf den Perfusionsbeutel wurde schrittweise externer Druck appliziert. Eine Niere wurde *in situ* über die Aorta perfundiert, die andere Niere wurde *ex situ* über die Nierenarterie perfundiert. Die Flussraten der Perfusion wurden unter den verschiedenen Druckstufen gemessen. Um den Surrogatparameter Fluss zu visualisieren, wurden gefärbte Mikropartikel (MPs; 10 μm) appliziert. Die Gefrierschnitte der Nieren wurden histologisch untersucht und die MPs quantifiziert.

**Ergebnisse**

*Die ex situ* Perfusion resultierte in einer signifikant erhöhten Flussrate (*P*<0.0001). Ferner war es unter der *ex situ* Perfusion durch eine zusätzliche Druckapplikation von 150 bis 200 mmHg möglich physiologische Flusswerte auf der Nierenarterie zu erreichen. Die gemessene Anzahl der MPs konnte den positiven Effekt der *ex situ* Perfusion auf die Mikrozirkulation im Nierenparenchym verdeutlichen (*P*<0.0001).

**Schlussfolgerungen**

Der Einsatz von MPs ermöglicht die Visualisierung der Nierenperfusion in einem experimentellen Versuchsaufbau. Die *ex situ* Perfusion der Niere bietet eine höhere Perfusionsqualität als die *in situ* Perfusion.

## Introduction

The literature on organ procurement is extensive, but the level of evidence provided is mainly low [1]. Static cold storage represents the present standard method of organ preservation. Starting with rapid vascular flush in order to remove residual blood components, the procedure also includes cooling as well as equilibration of the preservation solution with the conserved tissue [2]. Regarding multiorgan procurement, the perfusion of the grafts is performed via the aorta as so-called *in situ* perfusion [3], whereas isolated *ex situ* perfusion is only used in living-related kidney donation. Nevertheless, in multiorgan procurement, *ex situ* perfusion as a back-table procedure in addition to *in situ* perfusion may contribute to better rinsing of the graft and is advocated to ameliorate the perfusion quality [4]. To prove the advantages of *ex situ* perfusion we initiated the present trial. The kidney, being a paired organ, offers a unique opportunity to draw a comparison of the two different methods of *ex situ* versus *in situ* perfusion in one species being exposed to the same conditions. In order to visualise the efficiency of *ex situ* perfusion and the distribution of perfusate fluid in the target tissue, we applied different coloured microparticles (MPs). The correlation of perfusate flow in the renal artery (RA) and its effects on the microcirculation of the kidney parenchyma were analysed by this procedure.

## Materials and methods

Multiorgan procurement of 15 experimental animals (German landrace pigs; Herr B. Büttner, Buchenhof, 55270 Zornheim, Germany) was performed as described by Starzl and colleagues [3]. In order to align the conditions to those of prior experiments on the porcine liver, presented by our group [5], perfusion of kidney grafts was carried out using histidine–tryptophan–ketoglutarate (HTK). One kidney was perfused *in situ* via the aorta, whereas the second kidney received sole *ex situ* perfusion via the RA. Only one method of perfusion was therefore applied to each of the two organs, neither of them received both methods consecutively.

### Animals

The study was designed according to the guidelines of the German animal protection law and was approved by the local committee for animal welfare under the title ‘Perfusionsversuche im Rahmen einer Multi-Organentnahme beim Schwein’.

### Anaesthesia

The 15 experimental animals were premedicated by intramuscular administration of the sedative azaperone (7.5 mg/kg). Anaesthesia was initiated with an intravenous sodium thiopental bolus (5 mg/kg) and was maintained by intravenous infusion (10 mg/kg/hour). After intubation, pigs were mechanically ventilated with a Dräger respirator Servo 900b. Arterial and central venous lines were introduced via the femoral artery and vein. Prior to surgery, a 7.5 mg bolus of the analgesic piritramid was administered intravenously and maintained by intravenous infusion (0.25 mg/kg/hour). The heart rate and oxygen saturation were continuously measured using electrocardiography, pulse oximetry and capnometry. Ventilation was adjusted according to repeated blood gas analysis. For volume substitution, Ringer’s solution at 10 ml/kg was constantly administered during the operation. The experimental procedure was kept constant throughout the set of experiments as described previously by our group [5].

### Surgery

In this trial the multiorgan procurement technique was based on the method described by Starzl and colleagues [3,4]. The abdominal cavity was opened by a midline incision. The RA and the distal aorta were exposed by dissection of the retroperitoneum. An ultrasound probe was placed on the aorta as well as the RA in order to determine the baseline flow measurement indicating the physiological values of flow. After administration of 10,000 IU heparin, one kidney was explanted and *ex situ* perfusion was performed immediately as a back-table procedure. The explanted kidney was cooled down by pouring on ice-cold Ringer’s solution. An ultrasound probe was placed on the RA to measure the arterial flow during *ex situ* perfusion. The flow inside the artery was increased by addition of external pressure applied to the solution bag and slowly increased, taking records of flow measurements at different pressure steps. A bolus of blue MPs (2 ml and 100 ml NaCl) was subsequently administered to the RA through the same perfusion system.

In preparation of the *in situ* perfusion of the remaining kidney, the common iliac arteries and inferior mesenteric artery were ligated. An 18 Charrière perfusion cannula was placed into the distal aorta and connected to the perfusion bag via a transfusion system of 9 Charrière (200 μm filter, 175 cm, B93; Codan, Lensahn, Germany). Ultrasound probes were used to measure the arterial flow inside the aorta and the RA during the procedure. Cardiac arrest was induced by intravenous KCl application. A clamp was placed on the thoracic aorta at the level of diaphragm in order to prevent the solution from receding cranially (cross-clamp). Immediately after cardiac arrest, organs and operative findings were cooled down by pouring ice-cold Ringer’s solution into the abdominal cavity. After completing the *in situ* perfusion and administering red MPs (2 ml and 100 ml NaCl) the kidney was eventually explanted.

### Organ perfusion

**Figure 1:**
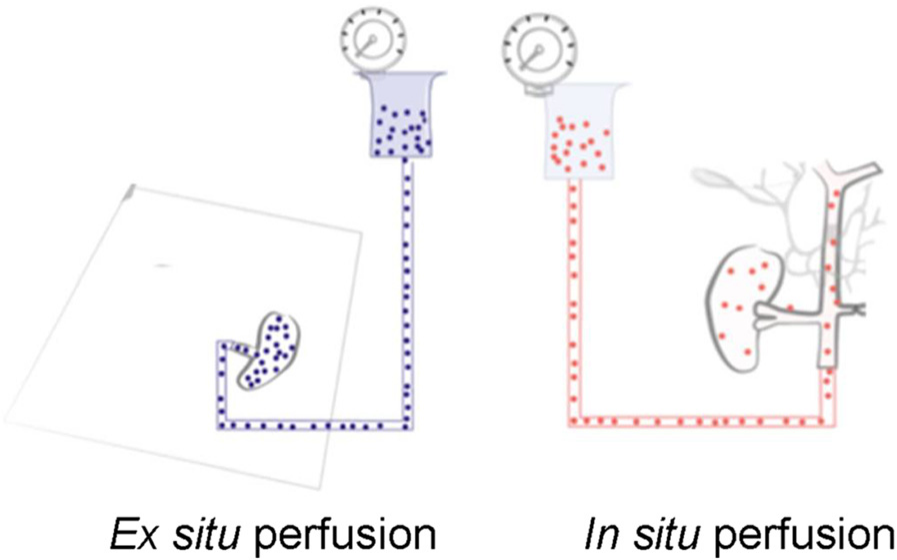
**Experimental setting of *in situ *perfusion (aorta) and *ex situ *perfusion (renal artery).** Red microparticles were used for *in situ* perfusion and blue microparticles for *ex situ* perfusion.

*Ex situ* perfusion was carried out via the RA (Figure 1). After nephrectomy, a transfusion system of 9 Charrière (200 μm filter, 175 cm, B93; Codan, Lensahn, Germany) was inserted and fixed into the RA. The explanted kidney was transferred into a bowl containing ice-cold Ringer’s solution to maintain the temperature at 4°C. *Ex situ* perfusion was performed at gravity pressure (100 mmHg) before adding external pressure to the preservation solution. This additional pressure was subsequently increased at intervals of 50 mmHg using a pressure gauge. The following intervals were chosen: gravity flow (100 mmHg), +50 (150 mmHg), +100 (200 mmHg), +150 (250 mmHg) and +200 (300 mmHg). The rate of flow inside the RA was measured during the procedure of pressure elevation. After *ex situ* perfusion, 100 ml NaCl containing blue-coloured MPs were infused into the kidney at gravity flow (100 mmHg).

*In situ* perfusion was carried out via the aortic cannula using cooled HTK solution (Figure 1). A gravity pressure of 100 mmHg was used. Flow measurements of the aorta and the RA were recorded. As described above, the pressure on the preservation solution was increased stepwise, adding pressure to the solution bag using a pressure gauge. The following pressure intervals were taken into account: gravity flow (100 mmHg), +50 (150 mmHg), +100 (200 mmHg), +150 (250 mmHg) and +200 (300 mmHg), as described previously by our group [5]. Subsequent to *in situ* perfusion, 100 ml NaCl containing a constant amount of 2 ml red-coloured MPs were infused at gravity flow.

### Microparticles

Additional data about the microcirculation of kidney parenchyma were obtained using MPs after *in situ* and *ex situ* perfusion. Samples of the kidneys were required and submitted for histomorphological examination. Non-radioactive red and blue MPs (10 μm) based on polystyrene were purchased from Sigma-Aldrich (St Louis, MO, USA). Prior to injection, 2 ml MPs were suspended in 100 ml normal saline suspension (NaCl 0.9%), vortexed and sonicated, to prevent the formation of MP aggregates.

Subsequently, the saline suspension (NaCl 0.9%) containing approximately the same amount of MPs (red or blue) was infused via the aorta after *in situ* perfusion (red) or via the RA after *ex situ* perfusion (blue) at constant and identical pressure of 100 mmHg (gravity flow).

### Histological examination

Tissue samples were quick frozen for cryotomic preparation, followed by performing 4 μm serial sections for microscopic and morphometric analysis [6]. Slides were stained by haematoxylin. Tissue slides were then examined by a Leica microscope (type DMLB; Leitz, Wetzlar, Germany). For histological examination, 10 representative high-power fields (0.302 mm^2^) were counted. The resulting amounts of these 10 microscopic fields were averaged.

### Statistical analysis

For descriptive analysis, the mean and standard deviation are presented. Box plots were used for graphic representation of the results. For evaluation of statistical significant differences for confirmatory analysis of flow inside the RA and the aorta at the different pressure steps, a simple two-tailed paired *t-*test was performed. To estimate the overall *P* value, a mixed linear model was used with pressure as the fixed effect and *in situ*/*ex situ* and the animal as the random effects.

The outcome flow rate was not normally distributed. We transformed the rate by the logarithm so that *t-*tests and a linear mixed model could be performed with the log-transformed variables.

The global significance level for all statistical test procedures conducted was chosen as α = 0.05. Due to multiple testing, Bonferroni correction (α/*n*, where *n* is the number of hypotheses, which are analysed as confirmatory) was performed. Fourteen hypotheses were tested, which results in 14 statistical tests. Every test was performed to the local significance level of 0.0035 (= 0.05/14). Only *P* <0.0035 is therefore considered statistically significant.

## Results

For this analysis 15 experimental animals were taken into account. The median weight was 32.3 kg (29.2 to 35.4 kg). Hemodynamic parameters were monitored and kept at a constant level until the point of perfusion. All animals survived anaesthesia and surgical interventions until perfusion. Table 1 displays the mean physiological values of the heart-beating animals prior to perfusion.

**Table 1:** Baseline measurements of pressure and flow under physiological conditions

	**Pressure aorta (mmHg)**	**Flow aorta (ml/minute)**	**Flow renal artery (ml/minute)**
Mean values of heart-beating pigs under physiological conditions prior to perfusion	86.6 (± 13.26)	789.13 (± 328.53)	168.47 (± 59.15)

**Figure 2:**
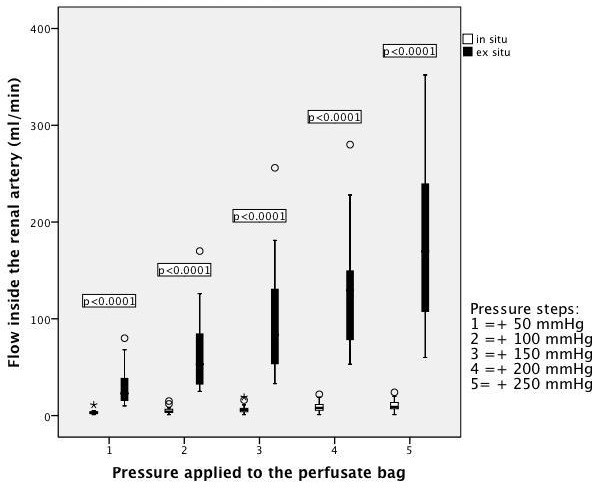
**Renal artery flow rates for *in situ *** **perfusion (white boxes) versus *ex situ *** **perfusion (black boxes) at different pressures.**

Figure 2 shows the flow rates at different steps of externally applied pressure for *in situ* perfusion (white boxes) in comparison with *ex situ* perfusion (black boxes). The statistical analysis of flow inside the RA revealed significant *P* values (*P* <0.0001) at each different pressure step (+50 mmHg, +100 mmHg, +150 mmHg, +200 mmHg), as displayed in Table 2. Overall *P* values for flow during *in situ* perfusion versus *ex situ* perfusion could also be proven as being highly significant (*P* <0.0001). Although the interaction of pressure application is significant for either *in situ* or *ex situ* perfusion, the box plot diagram displays that the flow does not change to a great extent when applying *in situ* perfusion (Figure 2).

**Table 2:** **Renal artery flow rates during *in situ *** **perfusion versus *ex situ *** **perfusion at different pressures**

	**Gravity flow (100 mmHg)**	**+50 mmHg (150 mmHg)**	**+100 mmHg (200 mmHg)**	**+150 mmHg (250 mmHg)**	**+200 mmHg (300 mmHg)**
Mean flow in renal artery during *in situ* perfusion (ml/minute)	3.47 (± 2.25)	5.21 (±3.97)	7.00 (± 5.04)	8.5 (± 5.92)	9.93 (± 6.29)
Mean flow in renal artery during *ex situ* perfusion (ml/minute)	30.78 (± 21.06)	68.85 (±46.38)	100.15 (± 65.19)	132.38 (± 68.01)	184.62 (± 85.94)
*P* value (*in situ* versus *ex situ*)	<0.0001	<0.0001	<0.0001	<0.0001	<0.0001

**Figure 3:**
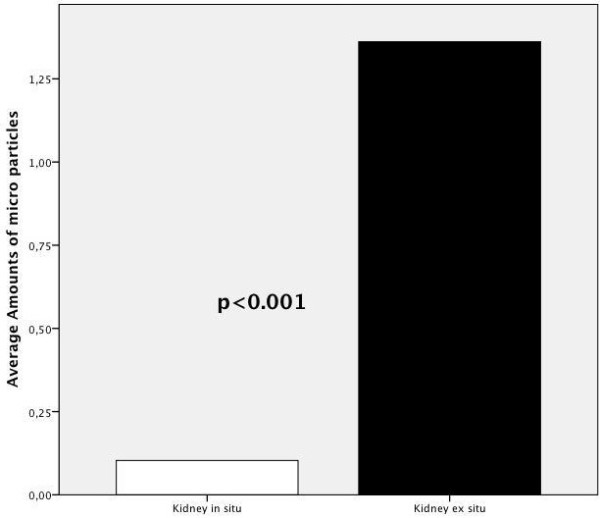
**Average amounts of microparticles trapped in kidney tissue after *in situ *** **perfusion (white) versus *ex situ *** **perfusion (black).**

Considering the bar chart for the number of MPs found in the frozen sections (Figure 3), it again becomes obvious in this respect that *ex situ* perfusion seems to be more efficient than *in situ* perfusion (100% vs. 8%). At any of the different locations of tissue sampling, the numbers of MPs originating from *ex situ* perfusion (black) was significantly higher (*P*<0.001) than those of *in situ* perfusion (white).

**Figure 4:**
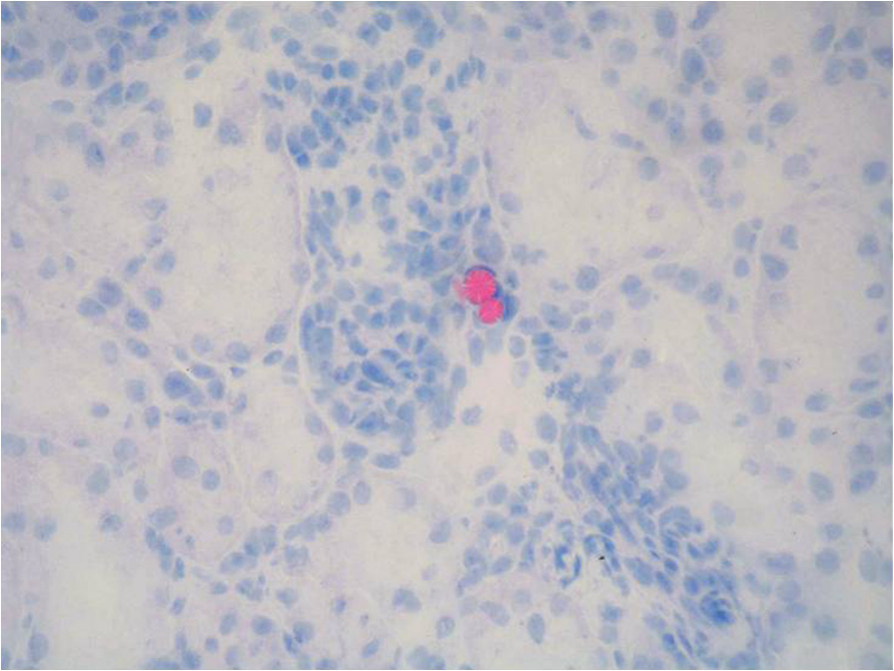
**High-power field (0.302 mm**^**2**^**) of kidney tissue showing microparticles being trapped after *in situ *** **perfusion (stained red).**

**Figure 5:**
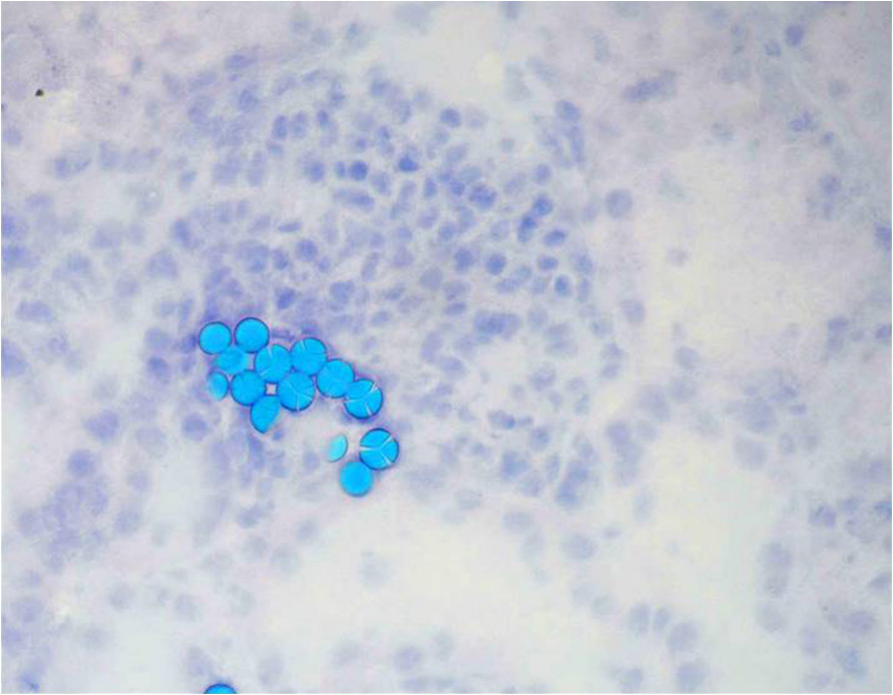
**High-power field (0.302 mm**^**2**^**) of kidney tissue showing microparticles being trapped after *ex situ *** **perfusion (stained blue).**

In general, the MPs were found in small capillaries of the kidney. Representative high-power fields of the kidney parenchyma after *in situ* perfusion and *ex situ* perfusion are presented in Figures 4 and 5, respectively. In general, most of the particles have been found in the glomerula, while there were fewer particles in capillaries surrounding the tubuli. By quantitative histological analysis, significantly more *ex situ* perfusion particles than *in situ* perfusion beats (*P*<0.001) could be observed. Microscopically, we could not demonstrate any specific differences between the two abovementioned groups.

## Discussion

In the standard procedure of multiorgan procurement, organ preservation is carried out via the aorta of the donor [3,4]. This so-called *in situ* perfusion can be improved by increasing the pressure on the solution bag [7]. In clinical settings of kidney procurement using *in situ* perfusion, an additional brief *ex situ* flush was recommended [8]. This is in line with the DTG procurement guidelines, stating that an additional *ex situ* pressure perfusion is advocated in order to check for clear flush and potential vascular injuries [4].

The present animal trial was initiated to draw a comparison between *ex situ* and *in situ* perfusion focusing on the perfusion flow and its impact on the microcirculation of the kidney graft. Since the rate of flow in organ procurement is only a surrogate parameter, we decided to additionally apply MPs as a visual medium in order to prove the effectiveness of *ex situ* perfusion. The diameter of MPs required was determined based on former experimental setups using MPs in animal models [6,9-13]: the quantification process of MPs might, however, be a possible source of error. In order to reduce the extent of this error, 10 microscopic views of each kidney sample were counted and the average values were taken into account. We considered HTK in this experimental setup, since it represents the current standard solution in abdominal multiorgan procurement in Germany.

Flow inside the RA was significantly improved by *ex situ* perfusion (*P*= 0.0001). Moreover, *ex situ* pressure perfusion resulted in significantly higher flow values compared with *in situ* pressure perfusion (*P* = 0.0001). This may also be explained by the physiological characteristics of the cardiovascular system, being a closed, elastic system filled with fluid. In this system, a positive mean cardiovascular pressure is prevalent, which is a result of elasticity and volume. A change in mean cardiovascular pressure occurs, whenever either the elasticity or volume inside the system is altered. Applying this principle in terms of multiorgan procurement, there is an enormous loss of blood volume due to incision of the vena cava and a loss of vessel tonus due to the occurrence of brain death. In contrast, the resistance inside the kidney circulation is increased due to vasoconstriction of RA induced by hypothermia [14]. These effects result in a loss of mean cardiovascular pressure, which has to be outweighed by the perfusate flow.

The aorta is a large, rigid and multiply branched vessel providing many options for losing intravascular pressure and flow. *In situ* perfusion can therefore quite conceivably not provide the requirements to build up an adequate perfusion flow since in this case the driving pressure is considerably low [12]. In contrast to this, *ex situ* perfusion is independent of any aortic characteristics. The application of perfusate flow directly into the RA (*ex situ*) does not provide any possibilities of loss in perfusion pressure and flow. In conclusion, *ex situ* perfusion is able to rinse the vascular system of the kidney more efficiently.

The additional application of MPs was able to confirm the findings of our surrogate parameter (flow). The application of a constant volume of 100 ml normal saline suspension (NaCl 0.9%) containing equal amounts of MPs for both treatment groups revealed that 100% of MPs were trapped inside the kidney receiving *ex situ* perfusion whereas only small amounts (8%) reached the target tissue by *in situ* perfusion. This indicates a loss of perfusion solution into other regions of the body when applying *in situ* perfusion [12].

The upper limit of pressure and flow is hard to define, as possible injuries due to sheer stress were not analysed in our model and kidneys were not transplanted. In summary, physiological flow rates can be achieved using *ex situ* pressure perfusion of 150 to 200 mmHg. However, considering *in situ* high-pressure perfusion, a prospective, randomised kidney transplant trial did not reveal any significant advantages for kidney graft survival, although the grafts obtaining high pressure perfusion did not show any primary non-function compared with a rate of 10.5% for organs perfused by gravity flow [15].

## Conclusions

The use of MPs represents a valuable method for visualising the quality of organ perfusion in experimental setups. *Ex situ* perfusion of the kidney grafts during multiorgan procurement results in significantly higher arterial flow rates than *in situ* perfusion. Furthermore, additional pressure perfusion was able to achieve significantly higher flow rates during *ex situ* perfusion compared with *in situ* perfusion. In conclusion, there is strong evidence that additional *ex situ* perfusion during kidney procurement is able to improve the quality of organ perfusion. The application of additional *ex situ* perfusion should therefore be advocated in kidney procurement.

## Abbreviations

HTK: Histidine–tryptophan–ketoglutarate; MP: Microparticle (10 μm, coloured red or blue); RA: Renal artery.

## Competing interests

The authors declare that they have no competing interests.

## Authors’ contributions

DF was responsible for the experimental design and execution, research and authoring. MK was responsible for research, data collection and support for preparation of the manuscript. MS was responsible for research, data collection, statistical analysis, authoring and proof reading. US was responsible for research, data collection and support for preparation of the manuscript. AH was responsible for anaesthesia and perioperative management of experimental animals. VW was responsible for statistical analysis. TH was responsible for histological examination. OK and GO were responsible for critical review of the manuscript. All authors read and approved the final version of the manuscript.

## Authors’ information

Data are part of the MD thesis of MS.
